# Pregnant women's knowledge of birth defects and their associated factors among antenatal care attendees in referral hospitals of Amhara regional state, Ethiopia, in 2019

**DOI:** 10.3389/fgwh.2023.1085645

**Published:** 2023-07-28

**Authors:** Addisu Andualem Ferede, Belayneh Ayanaw Kassie, Kiber Temesgen Mosu, Worku Taye Getahun, Birhan Tsegaw Taye, Melaku Desta, Mamaru Getie Fetene

**Affiliations:** ^1^ Department of Midwifery, College of Health Sciences, Debre Markos University, Debre Markos, Ethiopia; ^2^ Department of Clinical Midwifery, School of Midwifery, College of Medicine and Health Sciences, University of Gondar, Gondar, Ethiopia; ^3^ Department of Obstetrics and Gynecology, Debre Markos Comprehensive Specialized Hospital, Debre Markos, Ethiopia; ^4^ School of Nursing and Midwifery, Asrat Woldeyes Health Sciences Campus, Debre Berhan University, Debre Berhan, Ethiopia

**Keywords:** antenatal care, birth defects, knowledge, pregnant women, Ethiopia

## Abstract

**Background:**

Birth defects (BDs) are structural, behavioral, functional, and metabolic disorders present at birth. Due to lack of knowledge, families and communities stigmatized pregnant women following the birth of a child with birth defects. In Ethiopia, there was limited evidence to assess the level of knowledge among pregnant women despite increasing magnitude of birth defects.

**Objectives:**

This study aims to assess pregnant women's knowledge of birth defects and its associated factors among antenatal care (ANC) attendees in referral hospitals of Amhara regional state in 2019.

**Materials and methods:**

Between 1 June and 30 June 2019, 636 pregnant women receiving prenatal care participated in an institution-based cross-sectional study. The approach for sampling was multistage. A semi-structured pretested interviewer-administered questionnaire was used to collect data. Data were entered in EpiData version 4.6 and analyzed using SPSS version 25 software. A bivariable and multivariable logistic regression model was used. Odds ratio with 95% confidence interval and *p*-value of ≤0.05 declared statistical significance association.

**Results:**

A total of 636 pregnant women were included in the analysis. Accordingly, pregnant women's knowledge of birth defects was found to be 49.2% (95% CI: 45.4–53.1). Age group of <25 years (AOR = 0.16, 95% CI: 0.04–0.61), urban residence (AOR = 6.06, 95% CI: 2.17–16.94), ANC booked before 20 weeks of gestational age (AOR = 3.42, 95% CI: 1.37–8.54), and ever heard on birth defects (AOR = 5.00, 95% CI: 1.87–13.43) were significantly associated factors with pregnant women's knowledge of birth defects.

**Conclusions:**

Approximately half of the pregnant mothers were aware of birth defects. Addressing pre-pregnancy and pregnancy health information and education particularly on the prevention of birth defects is recommended.

## Introduction

Birth defects (BDs) are structural, cognitive, or functional problems that develop during pregnancy and can be detected before, during, or after birth (1). Despite causing miscarriage and stillbirth, BDs are an under-recognized cause of infant mortality and disability in children (2). The World Health Organization (WHO) estimates that BDs cause 260,000 deaths, accounting for approximately 7% of all neonatal deaths (3). Every year, more than 8 million babies are born with BDs worldwide, and the number of BDs is increasing (4, 5). Interventions during pregnancy, such as taking folic acid, iodine, and iron, and raising awareness about the effects of teratogen exposure will significantly reduce the number of BDs (6).

The regions of Africa that account for 5.2%–75% BDs are in middle- and low-income countries with exposure to infection, teratogen, and poor maternal nutrient intake (7–9). Early identification or recognition of causative and risk factors for preventing BDs by involving women is essential due to public health significance in the high cost of treatment and rehabilitation activities (10, 11).

Folic acid is advised for women to take to lessen the load of BDs using proper strategies and education (12). More than 90% of newborns delivered with serious BDs are in middle- and low-income nations, where there are inadequate services for their under-five patients, which causes the majority of them to pass away at a young age (13). Families with BD children face both financial and emotional difficulties (14).

Despite the burden of the problem and the role of mothers in the primary prevention of anomalies, pregnant women still lacked basic understanding about BDs (15). Knowing about BDs, their contributing factors, preventative measures, and treatment alternatives would ease the burden on the family, the nation, and the economy (16).

The morbidity and mortality of the child decreased by involving preventive care plan and interventions when mothers have sufficient understanding of BDs (17). Most women in Kenya who gave birth to BD children were ignored, did not receive care, were blamed and stigmatized because they did not know enough about how to take care of them, and were hidden from the community (18).

Studies in Ethiopia also found that the prevalence of BDs is high (19). The studies’ primary goal was to demonstrate the magnitude of BDs using retrospective data, rather than to raise awareness among pregnant women (20). In the different regions of the country, folic acid supplementation for the prevention of neural tube defects is uncommon; as a result, the magnitude of BDs has recently increased from 1.14% to 2.83% (21, 22). Women in Ethiopia have limited knowledge of preconception care that can help prevent BDs (23). Even medical professionals did not fully comprehend the safety of drugs during pregnancy (whether they cause BDs or not) (24).

Previous studies in Ethiopia focused on the prevalence of BDs by analyzing secondary data which misses the knowledge level of pregnant women that may not be sufficient to reduce the burden of BDs on the family, the community, and the country. Similarly, secondary data might not include some key variables related to the knowledge level of pregnant women about BDs. Therefore, this study was aimed to assess pregnant mothers’ knowledge of BDs and associated factors among antenatal care (ANC) attendees in selected referral hospitals of Amhara regional state, Ethiopia.

## Materials and methods

### Study design, setting, and period

The institutional-based cross-sectional study was conducted between 1 June and 30 June 2019. This study was conducted in referral hospitals of the Amhara regional state. Amhara region is one of the 10 Ethiopian states in the country located in the northwest part of the capital city of the country Addis Ababa. Twenty-eight million people live in the catchment area of this region, with roughly equal numbers of males and females. The current study was conducted at hospitals. Approximately 81 hospitals (6 referrals, 2 general, and 73 primary hospitals), 847 health facilities, and 3,342 health posts are located in the Amhara regional state. The six referral hospitals are named as the University of Gondar, Tibebe Ghion, Felege Hiwot, Debre Berhan, and Debre Markos. Each hospital was assumed to serve 4 million people, and 490–550 pregnant women were assumed to attend ANC service every month in each referral hospital. According to the data taken from the ANC registration book of the three hospitals, Debre Berhan Hospital provides annual ANC for 5,880 pregnant women. Furthermore, Debre Markos Hospital provides annual ANC for 6,240 pregnant women. Moreover, the University of Gondar Hospital gives annual ANC to 6,600 pregnant women.

### Population

The source of the population included all pregnant women who attended antenatal care in Amhara regional state referral hospitals. The study included all pregnant women who attended ANC during the data collection time in randomly selected hospitals. Pregnant women with communication problems like having hearing impairment or critically ill were excluded.

### Sample size determination

Sample size was determined by single population proportion formula by taking 50% proportion, 95% confidence level, 5% margin of error, and 1.5 design effect and adding 10% non-response rate.

The formula to determine the sample size was as follows:

$$n=\frac{{\left(Z\alpha /2\right)}^{2}p(1-p)}{d^{2}}(n)=\frac{{\left(1.96\right)}^{2*}0.5(1-0.5)}{(0.05)2}=385$$

By considering 10% non-response rate and 1.5 design effect, the final sample size became 636.

### Sampling technique and procedure

The approach for sampling was multistage. Six referral hospitals in the Amhara region at the time of the study were identified. Out of these, three of them were selected by simple random sampling using the lottery method. These were Debre Berhan referral hospital, Debre Markos referral hospital, and the University of Gondar teaching referral hospital. Based on the case flow, the total sample size was proportionally distributed to each chosen referral hospital. In accordance with the most recent 1-month report, the flow of pregnant women attending ANC services in the selected hospitals was summarized. Each selected referral hospital provided ANC service to 490–550 pregnant women per month. Then, systematic random sampling was employed to take the study participants in each referral hospital. The interval size “*k*” was taken by dividing the total population based on the last 1-month report of 1,560 by the sample size of 636 (1,560/636 = 2.45). Finally, every third pregnant woman was taken after randomly selecting the first sample from 1 to 3.

### Measurements and operational definitions

#### Independent variables

##### Socio-demographic variables

The socio-demographic variables were age, residence, religion, maternal educational level, maternal occupation, marital status, husband’s educational level, husband’s occupation, and average monthly family income.

##### Obstetric variables

The obstetric variables were reported to be gravidity, parity, number of children, prior abortion, prior stillbirth, and prior birth defect.

##### Service-related variables

The service-related variables were the number of ANC visit, ANC booking time, uptake of iron folate, and having ever heard about BDs.

### Dependent variable

The dependent variable was knowledge of birth defects among pregnant women. Knowledge was assessed by asking the participants 20 “Yes” or “No” item questions. One point was given for all the correct answers, while zero point for incorrect answers. Then, women's knowledge was composed (knowledgeable which was coded as “1” and not knowing which was coded as “0”). The tool was developed from a validated document of previous studies and modified in to the local context.

**Good knowledge**: mothers who scored 50% and above for knowledge assessment questions had good knowledge (25).

**Birth defects**: BDs are anomalies in the structure, function, or mental development that develop during pregnancy and can be detected before, during, or after birth (26).

**Folic acid taken**: women who took folic acid supplement at least 1 month before pregnancy and continued for 3 months after pregnancy (21).

### Data collection tools and procedures

Data were acquired from pregnant women using a face-to-face exit interview with a semi-structured questionnaire. The questionnaire included knowledge assessment questions and questions about socio-demographic, obstetrical, and service-related factors. The questionnaire was developed using variables that were modified from the different literature (15, 16, 27). Experts who were health educators in pediatrics assessed the content validity of the tool, and a correction was made on the clarity of sentences, appropriateness of content, and sequence of items. Cronbach's alpha (*α* = 0.77) was calculated for the knowledge assessment questions to determine reliability. The following key steps were used to compute the test using SPSS software. After the SPSS is opened, we clicked the analyses, then went down to the scale, and got a reliability analysis. When we clicked the reliability analysis, the model alpha appeared. At this time, select, drag, and drop the knowledge assessment questions into the open field. After pressing the “OK” button, the result for Cronbach's alpha was displayed. Three MSc and six BSc midwives were involved in the supervision and data collection procedures, respectively.

### Data quality control

Designing a data gathering tool with the goal of ensuring data quality was prioritized. The questionnaire was prepared in the English language first, then translated into the local language Amharic, and finally changed back to the English language again to keep its consistency. The pretest was done on 5% (32) of the total sample size at Felege Hiwot Comprehensive Specialized Hospital before the actual data collection time. Before the data collection, the supervisors and data collectors received a 1-day training on the study's goals, importance, data gathering procedures, interviewing techniques, and how to approach and handle the participants’ personal information. Data collectors were supervised, and the questionnaire was evaluated daily to ensure that the interview was comprehensive, with corrections quickly addressed throughout the data gathering period.

### Data processing and analysis

After being double-checked, coded, and put into the EpiData version 4.6 software, the data were exported and then analyzed in SPSS version 25. The results of the finding were presented using texts, tables, frequency, and percentage. A multivariable logistic regression model was used to manage the impact of confounders on the outcome variable after using binary logistic regression to find the associated factors of BDs on knowledge. Variables that had a *p*-value of <0.25 in the bivariable analysis were entered into a multivariable logistic regression model. The 95% CI of odds ratio was computed. A *p*-value of less than 0.05 in the multivariable logistic regression analysis was taken as significance to determine associated factors of knowledge. Hosmer–Lemeshow goodness assessed the models’ fitness.

### Ethical consideration

The ethical review committee of the School of Midwifery gained authorization from the Institution Review Board of the University of Gondar, College of Medicine and Health Sciences (Ref No: MIDW/H34/09/2011), and the Amhara Regional Health Bureau was asked to issue a permission letter. The formal letter was sent to Amhara regional state referral hospitals, and a written approval letter was acquired from the study's governing agencies. The study participants were then told of the study's goal, the relevance of their involvement, and their freedom to refuse to participate or withdraw at any time. Before data collection, written informed consent was sought. The information provided by each respondent was kept private and discreet, and it was anonymous.

## Results

### Socio-demographic characteristics

Six hundred thirty-six pregnant women who attended ANC with a response rate of 100% were included in this study. The median age of respondents was 28 years (IQR ± 9) with a range of 19–48 years. The majority of the respondents, 550 (86.5%) and 614 (96.5%), were an orthodox followers and married, respectively (Table 1).

**Table 1:** Socio-demographic characteristics of pregnant women attending ANC in Amhara regional state selected referral hospitals in 2019 (*n* = 636).

Variables	Frequency	Percentage
Age		
<25	173	27.2
25–34	326	51.3
≥35	137	21.5
Residence		
Urban	540	84.9
Rural	96	15.1
Religion		
Orthodox	550	86.5
Muslim	55	8.6
Others^a^	31	4.9
Respondent education		
No formal education	146	23.0
Primary school	84	13.2
Secondary school	169	26.6
College and above	237	37.2
Respondent occupation		
Housewife	258	40.6
Government employee	169	26.5
Private employee	40	6.3
Farmer	54	8.5
Self-employed	115	18.1
Marital status		
Single	14	2.2
Married	614	96.5
Others^b^	8	1.3
Partner/husband education (*n* = 614)		
No formal education	33	5.4
Primary school	80	13.0
Secondary school	128	20.9
College and above	373	60.7
Partner/husband occupation (*n* = 614)		
Government	330	53.8
Private	115	18.7
Farmer	51	8.3
Self-employed	118	19.2
Average monthly income (Ethiopian Birr)		
≤1,000	77	12.1
1,001–3,000	309	48.6
3,001–4,999	112	17.6
≥5,000	138	21.7

^a^ Protestant, Catholic.

^b^ Divorced, widowed.

### Obstetrics characteristics

Of the respondents, 350 (55%) and 243 (56.9%) had two to four times pregnancies and births, respectively. From the respondents who had birth, 23 (5.4%) had a history of birth defect child (Table 2).

**Table 2:** Obstetrics-related characteristics of pregnant women attending ANC in Amhara regional state selected referral hospitals in 2019 (*n* = 636).

Variables	Frequency	Percentage
Gravidity		
1	209	32.9
2–4	350	55.0
≥5	77	12.1
Parity (*n* = 427)		
1	163	38.2
2–4	243	56.9
≥5	21	4.9
Number of children (*n* = 427)		
1	53	12.4
2	47	11.0
≥3	327	76.6
Stillbirth (*n* = 427)		
Yes	56	13.1
No	371	86.9
Defected child delivered (*n* = 427)		
Yes	23	5.4
No	404	94.6
Took folic acid to prevent the next occurrence (*n* = 23)		
Yes	7	30.4
No	16	69.6
Abortion (*n* = 427)		
Yes	90	21.1
No	337	78.9

### Service-related characteristics

Nearly half of the respondents (305, 48%) had two to three ANC visits. Respondents who heard on BDs (214, 40.7%) took the main source of information from their friends. Only 37 (11.2%) of the participants said that iron folate is given for the prevention of congenital anomaly (Table 3).

**Table 3:** Service-related characteristics of pregnant women attending ANC in Amhara regional state selected referral hospitals in 2019 (*n* = 636).

Variables	Frequency	Percentage
ANC booked time		
<20 weeks	517	81.3
≥20 weeks	119	18.7
ANC visit		
1	211	33.2
2–3	305	48.0
≥4	120	18.8
Took iron folate during ANC		
Yes	331	52.0
No	305	48.0
Reason to take iron folate (*n* = 331)		
Prevention of anemia		
Yes	275	83.1
No	56	16.9
Prevention of congenital anomaly		
Yes	37	11.2
No	294	88.8
Development of fetal growth		
Yes	33	10.0
No	298	90.0
Prevention of PPH		
Yes	85	25.7
No	246	74.3
Do not know	32	9.7
Having ever heard on BDs		
Yes	538	84.6
No	98	15.4
Source of information		
During ANC follow-up		
Yes	271	42.6
No	365	57.4
Mass media		
Yes	170	31.6
No	368	68.4
Friends		
Yes	219	40.7
No	319	59.3
Relatives		
Yes	52	9.7
No	486	90.3
Social media		
Yes	74	13.8
No	464	86.2

### Knowledge of the respondents

From 636 study participants, 313 (49.2%) had good knowledge, while 323 (50.8%) had poor knowledge. The mean overall knowledge of pregnant women was 9.87. From 20 knowledge assessment questions, the minimum and maximum answer of the respondents was 0 and 18, respectively. Almost three-fifths (377, 59.3%) of pregnant women knew about BDs to occur during fetal life. One-quarter (159, 25%) of the study participants said that physiotherapy can be used as a management of BDs. Two-thirds (421, 66.2%) of respondents knew that smoking during pregnancy brings BDs. More than three-fourths of the respondents (490, 77%) knew that BDs can cause psychological stress (Table 4).

**Table 4:** Respondents' knowledge of birth defects among antenatal care attendees in Amhara regional state selected referral hospitals in 2019 (*n* = 636).

Items	Yes *n* (%)	No *n* (%)
BDs occur during fetal life	377 (59.3)	259 (40.7)
It can cause psychological stress	490 (77)	146 (23)
It can be detected later as well as after birth	377 (59.3)	259 (40.7)
BDs inherited through consanguinity of parents	347 (54.6)	289 (45.4)
Age more than 40-year results of birth defects	204 (32.1)	432 (67.9)
Obesity of the mother brings birth defects	168 (26.4)	468 (73.6)
BDs occur due to the chronic illness of the mother	343 (53.9)	293 (46.1)
BDs occur due to infection of the mother	391 (61.5)	245 (38.5)
Medication taken during pregnancy without prescription of health provider result in BDs	425 (66.8)	211 (33.2)
Smoking during pregnancy brings BDs	421 (66.2)	215 (33.8)
Alcohol consumption during pregnancy brings BDs	362 (56.9)	274 (43.1)
Exposure to radiation during pregnancy brings BDs	241 (37.9)	395 (62.1)
Supernatural factors bring BDs	296 (46.5)	340 (53.5)
Being contacted with infected dogs or cats feces during pregnancy results in birth defects	220 (34.6)	416 (65.4)
Many birth defects are preventable	343 (53.9)	293 (46.1)
Intake of iodized salt reduces the risk of BDs	368 (57.9)	268 (42.1)
Preconception folic acid intake prevents the occurrence	397 (62.4)	239 (37.6)
BDs can be treatable with medical management	343 (53.9)	293 (46.1)
BDs can be treatable with surgical management	223 (35.1)	413 (64.9)
Physiotherapy used in the management of BDs	159 (25)	477 (75)

### Factors associated with women's knowledge of birth defects

A binary logistic regression analysis showed that age, residence, respondent and partner education, number of children, child born with defect, ANC booked time, number of ANC visit, the uptake of iron folate, and having ever heard about BDs were all significantly associated with knowledge of BDs among pregnant women attending ANC.

However, on multivariable analysis, age, residence, ANC booked time, and having ever heard about BDs were identified as independently significant associated factors with pregnant women's knowledge of BDs.

According to this study, pregnant women who were under the age of 25 were 84% less likely to have adequate knowledge about BDs than pregnant women who were over the age of 35 years (AOR = 0.16, 95% CI: 0.04–0.61).

In the current study, the odds of having good knowledge of BDs among urban reside pregnant women were 6.06 times than those who reside in the rural area (AOR = 6.06, 95% CI: 2.17–16.94).

This study also found that the odds of having good knowledge of BDs among pregnant women who booked ANC before 20 weeks were 3.42 times more likely than those who booked in the late gestational age (AOR = 3.42, 95% CI: 1.37–8.54).

Moreover, the present study revealed that the odds of having good knowledge of BDs among pregnant women who have ever heard about BDs were 5.00 times than not having ever heard (AOR = 5.00, 95% CI: 1.87–13.43) (Table 5).

**Table 5:** Bivariable and multivariable logistic regression analysis results of factors associated with knowledge of birth defects among pregnant women attending ANC in Amhara regional state selected referral hospitals in 2019 (*n* = 636).

Variables	Good knowledge	Poor knowledge	COR (95% CI)	AOR (95% CI)
Age				
<25	71	102	0.63 (0.40–0.99)	0.16 (0.04–0.61)^a^
25–34	170	156	0.98 (0.66–1.47)	0.42 (0.16–1.11)
≥35	72	65	1	1
Residence				
Urban	286	254	2.88 (1.79–4.63)	6.06 (2.17,16.94)^b^
Rural	27	69	1	1
Maternal education				
No formal education	60	86	0.63 (0.41–0.95)	1.66 (0.6–4.62)
Primary school	36	48	0.67 (0.41–1.11)	1.21 (0.45–3.32)
Secondary school	92	77	1.07 (0.72–1.59)	1.70 (0.7–4.15)
College and above	125	112	1	1
Partner education				
No formal education	10	23	0.44 (0.20–0.94)	0.76 (0.07–7.90)
Primary school	38	42	0.91 (0.56–1.48)	1.06 (0.34–3.29)
Secondary school	69	59	1.18 (0.79–1.76)	3.63 (0.32–40.86)
College and above	186	187	1	1
No. of children				
1	31	22	1.44 (0.80–2.58)	1.45 (0.59–3.57)
2	26	21	1.26 (0.68–2.33)	2.10 (0.87–5.07)
≥3	162	165	1	1
Prior defected child				
Yes	11	3	3.41 (0.94–12.0)	0.48 (0.14–1.69)
No	214	199	1	1
ANC booked time				
<20 weeks	267	250	1.7 (1.13–2.55)	3.42 (1.37–8.54)^a^
≥20 weeks	46	73	1	1
No. of ANC visits				
1	77	134	0.54 (0.34–0.85)	2.75 (0.91–8.33)
2–3	174	131	1.24 (0.81–1.9)	2.29 (0.95–5.52)
≥4	62	58	1	1
Took iron folate during ANC				
Yes	183	148	1.67 (1.22–2.28)	1.04 (0.51–2.12)
No	130	175	1	1
Having ever heard on BDs				
Yes	284	226	4.20 (2.68–6.59)	5.00 (1.87–13.43)^b^
No	29	97	1	1

^a^ *p*-value of <0.05.

^b^ *p*-value of <0.001.

## Discussion

This study was carried out to determine pregnant women knowledge of birth defects and its associated factors among ANC attendees in three referral hospitals of Amhara regional state, Ethiopia. Thus, overall, nearly half of the respondents were knowledgeable on birth defects.

The proportion of pregnant women who had good knowledge on birth defects among ANC attendees was found to be 313 (49.2%) with 95% CI of 45.4–53.1. This is in line with the study conducted in Saudi (51%) (25). However, this finding was lower than the study conducted in north Iran, 53.3% (15); Karad, India, 63.5% (17); Sri Lanka, 56.4% (16); and Egypt, 76% (25). The possible explanation for this difference might be due to variation in data collection method and participants’ level of education. The previous studies included only pregnant women who could read and understand a self-administered questionnaire of the local language that may maximize the response of the women. Once more, the majority of responders to this study were housewives who might not have access to information concerning BDs.

On the other hand, the proportion of good knowledge was higher than the study conducted in two different places of Nigeria [25.6% (28) and 19.8% (29)] and Ghana [42.6% (27)]. The reason for this difference might be due to the time gap. The present study was recently conducted as compared to other studies. As maternal health becomes more prominent on the national and global agendas, healthcare-seeking behavior, access to health institutions, and professional competence are likely to improve, resulting in improved knowledge of the BDs among pregnant women. Another reason for this variation might be that previous studies were used as the consecutive and convenience sampling method to collect the data. In addition, the study in Ghana was used as a self-administered technique in which pregnant women may not easily understand the questions. Furthermore, the difference might be due to the characteristics of the respondents. For instance, majority (84.2%) and (29%) of the participants in Nigeria were single and had no formal education, respectively, while the present study showed that 95.5% were married and 37.2% had college and above education.

Pregnant women who were in the age group of less than 25 years were 84% less likely to be knowledgeable about BDs as compared to the age range of ≥35 years. This was consistent with the studies done in Iran (15) and Nigeria (29). One possible explanation is that as the mother's age became higher and higher, she may gain more experience and the likelihood of having more information increases. On the other hand, pregnant mothers under the age of 25 years may be afraid of discussing BDs openly to obtain critical information.

Regarding residence, pregnant women who reside in the urban were 6.06 times more knowledgeable about BDs as compared with those residing in the rural area. This was supported by the study conducted in Karad, India (17). The reason could be that pregnant women who live in rural areas may not be exposed to varied media material, may not receive much attention, and may prioritize their jobs over their health needs.

Pregnant women who booked ANC before 20 weeks of gestation were 3.42 times more knowledgeable on BDs than those who came after ≥20 weeks of gestation. This was supported by the study done in Nigeria (28). The possible explanation for this may be because since most BDs develop before the halfway point of pregnancy and are easily detectable by ultrasound, pregnant women who attended their ANC follow-up appointments early may have received timely information and known more about the health of their unborn child.

In this study, pregnant women who had ever heard about BDs were 5.00 times more knowledgeable than those who have not heard about it. This was consistent with the study conducted in Sri Lanka (16). The possible explanation for this might be that whenever pregnant women have heard about BDs from different sources such as healthcare providers, friends, mass media, and relatives, they may share, discuss, and get an idea about the malformation. Moreover, health education given to pregnant mothers during ANC follow-up may allow reading further to dig out pregnancy-related complications and BDs.

The finding of this study also showed that majority (84.6%) of pregnant women had ever heard about BDs. The main sources of information were ANC (42.6%) and friends (40.7%). The study in Nigeria reported that 50.5% of the participants were having ever heard about BDs and the main source of information (62.4%) was family members (29).

However, pregnant women were asked about the reason why iron folate was taken. Most (83.1%) respondents knew about prevention of anemia. However, 11.2% and 10% of respondents correctly stated the importance of iron folate as prevention of BDs and for fetal development, respectively. The study in Sri Lanka was reported that 5% of pregnant women correctly stated that iron folate is given for the prevention of BDs, while 35.8% of them said it is for the development of the nervous system of the fetus (16).

The theoretical implication of this study was assessing pregnant women's knowledge and associated factors. It is an entry point for the prevention of many BDs and delayed interventions. Knowing about BDs is important for the betterment of the affected individuals and to alleviate the cost to the family.

## Strength and limitation

A substantial number of study locations were used in the study, which allowed researchers to generalize about how representative the population was. Nevertheless, this study had some limitations. Due to the nature of the study design, temporal relations could not be assessed. Since the information was gained after pregnant women had got their ANC service, social desirability bias could be introduced.

## Conclusion and recommendation

Based on the finding obtained in this study, 49.2% of pregnant women who attended ANC were aware of BDs. Women's knowledge on BDs was influenced by age, residence, ANC booked time, and having ever heard about BDs. Therefore, efforts should strengthen to increase knowledge of the issue of BDs among pregnant women through various media, especially in rural areas, by using radio airtime programs and by providing proper health education, particularly to young pregnant women, while also encouraging early ANC to follow up to provide basic information about BDs. Additional qualitative and community-based research on the knowledge of BDs among women in the reproductive age range would be advisable.

## Data Availability

The original contributions presented in the study are included in the article, further inquiries can be directed to the corresponding author.
